# A Soccer Shot with Lengthy Consequences—Case Report & Current Literature Review of Commotio Cordis

**DOI:** 10.3390/jcm12062323

**Published:** 2023-03-16

**Authors:** Philipp Spitaler, Markus Stühlinger, Agne Adukauskaite, Axel Bauer, Wolfgang Dichtl

**Affiliations:** Department of Internal Medicine III, Medical University of Innsbruck, 6020 Innsbruck, Austria

**Keywords:** commotio cordis, ventricular tachycardia, implantable cardioverter-defibrillator, epicardial ablation

## Abstract

(1) Background: Commotio cordis, caused by objects being directly delivered to the chest, may cause cardiac arrest in young athletes, even without identifiable structural damage to the sternum, ribs or heart itself. Its prevention and management often remain suboptimal, resulting in dismal outcomes. (2) Case summary: A 32-year semi-professional goalkeeper suffered from a non-penetrating blunt thoracic trauma after being struck by a high-velocity shot during a regional league soccer game. He immediately lost consciousness, collapsed, and was successfully resuscitated through early defibrillation of ventricular fibrillation. After an uneventful follow-up for approximately 6 years, recurrent episodes of ventricular tachycardia occurred, which could ultimately only be prevented by epicardial ablation. (3) Conclusion: Very late recurrences of ventricular tachyarrhythmias may occur after ventricular fibrillation due to blunt chest trauma, even in the primary absence of evident structural myocardial damage.

## 1. Case Presentation

A 32-year-old semi-professional goalkeeper suffered from a non-penetrating blunt thoracic trauma after being struck by a high-velocity shot during a regional league soccer game. This led to an immediate loss of consciousness and subsequent collapse. Prompt initiation of cardiopulmonary resuscitation was undertaken. The first ECG revealed ventricular fibrillation (VF) as the underlying rhythm. After three external electrical defibrillations, the patient had a return of spontaneous circulation with restoration of sinus rhythm. A total of 90 min after the incident, the patient arrived, hemodynamically stable, at the hospital.

## 2. Patient Information (De-Identified)

The patient had no comorbidities and a negative family history regarding sudden cardiac death (SCD) or other cardiovascular diseases. Furthermore, the patient did not have any pre-existing medication nor did he report any substance abuse. A timeline of major events is given in Table 1.

## 3. Physical Examination and Diagnostic Assessment

**Figure 1:**
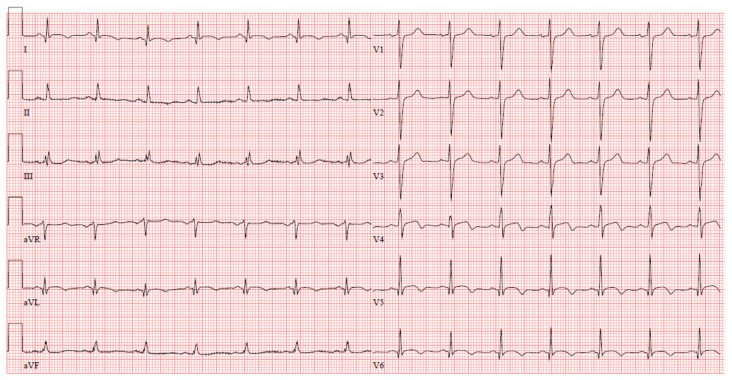
Initial electrocardiogram on admission to the hospital: sinus rhythm, ST-T abnormalities in V4–V6, I and aVL, left axis deviation, QTc 446 milliseconds.

On admission to the hospital, the initial electrocardiogram showed sinus rhythm with ST-T abnormalities in the precordial leads V4–V6 and the peripheral leads I and aVL, as well as left axis deviation. The corrected QT interval (QTc) measurement was 446 milliseconds (Figure 1). Notably, the ST-T abnormalities normalized in the subsequent 7 days.

Transthoracic echocardiography revealed normal ventricular dimensions, no regional wall motion abnormalities, and a mildly reduced LV global systolic function, which normalized in the following weeks.

**Figure 2:**
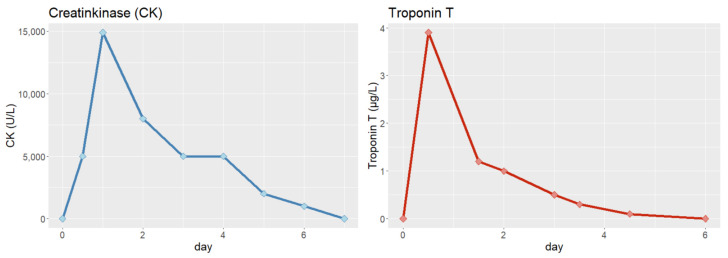
Trends of cardiac biomarkers during the first week after resuscitation.

Initial blood analysis revealed an elevated cardiac troponin-T of 0.199 µg/L (normal range, 0–0.039 µg/L), elevated creatine kinase of 656 U/L (normal range, 38–174 U/L), elevated lactate of 120 mg/dL (normal, range 5–20 mg/dL) and elevated D-dimer of 660 µg/L (normal range, 0–190 µg/L). Cardiac biomarkers normalized in the subsequent days (Figure 2). 

Coronary angiography demonstrated normal coronary arteries with no visible disease or luminal irregularities. Cardiac magnetic resonance (CMR) showed no evidence of arrhythmogenic right ventricular cardiomyopathy (ARVC). Cerebral CT and thoracic CT examinations were normal.

## 4. Differential Diagnosis

Considering the above-mentioned examinations, differential diagnosis included commotio cordis, myocardial infarction with non-obstructive coronary arteries (MINOCA), and any form of genetic channelopathy or primary ventricular fibrillation without early repolarization syndrome.

## 5. Interventions

During hospitalization, the patient remained free of further arrhythmias. In accordance with the 36th Bethesda conference of the Eligibility Recommendations for Competitive Athletes with Cardiovascular Abnormalities, a shared decision was taken with the patient and his family to implant an implantable cardioverter-defibrillator (ICD) through a transvenous approach (as subcutaneous ICDs were not available at the time), on day 10 after the incident. Prior to discharge, the patient was advised not to participate in any moderate- or high-intensity competitive sports, such as playing soccer, again [1].

## 6. Follow-Up

Due to recurrent inappropriate ICD shocks due to Riata^®^ ICD electrode malfunction within two years of initial presentation, a new ICD lead had to be performed without removal of the old one. This decision was based on the notion that Riata^®^ lead extraction is associated with a high rate of major complications (1 in every 80 procedures) and even mortality (1 death in every 166 procedures) [2,3].

**Figure 3:**
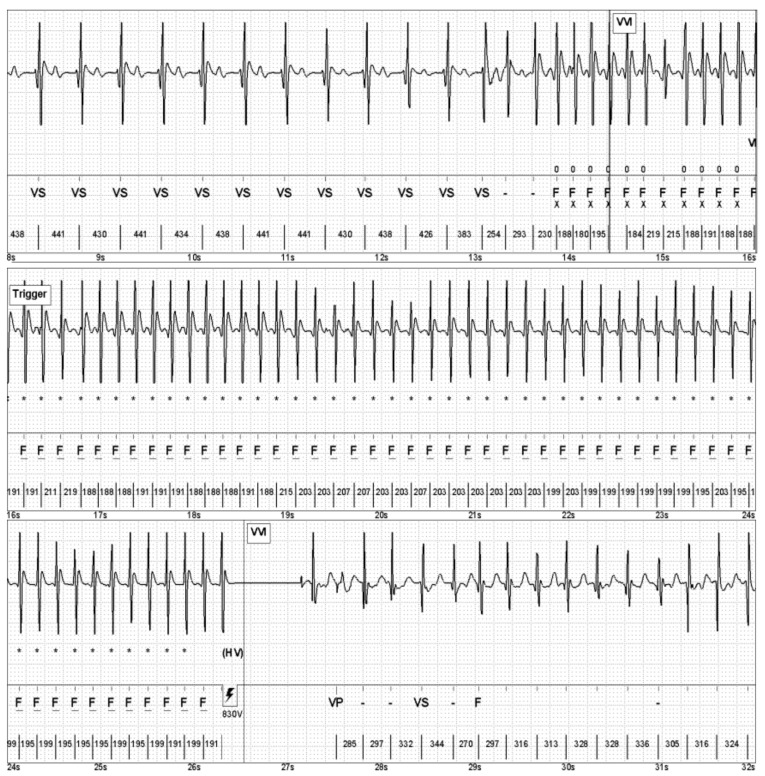
IEGM of a fast VT, appropriately terminated by shock delivery.

Six years after the index event, the patient suddenly felt dizzy upon quickly standing up in an indoor soccer game. Subsequently, an electrical shock was delivered by the ICD. In the following ICD readout, the intracardiac electrogram (IEGM) revealed a fast ventricular tachycardia (VT), appropriately terminated by an electrical shock (Figure 3).

Eleven years after the initial presentation, the patient suffered again from three appropriate shocks due to fast VTs. Hence, antiarrhythmic medication with amiodarone was started. Later, amiodarone was replaced by sotalol due to adverse effects. Due to the abandoned Riata^®^ ICD lead, another CMR scan unfortunately could not be performed at this timepoint. Since the patient had no atherosclerotic risk factors and no symptoms of angina pectoris, it was also decided not to repeat coronary angiography.

**Figure 4:**
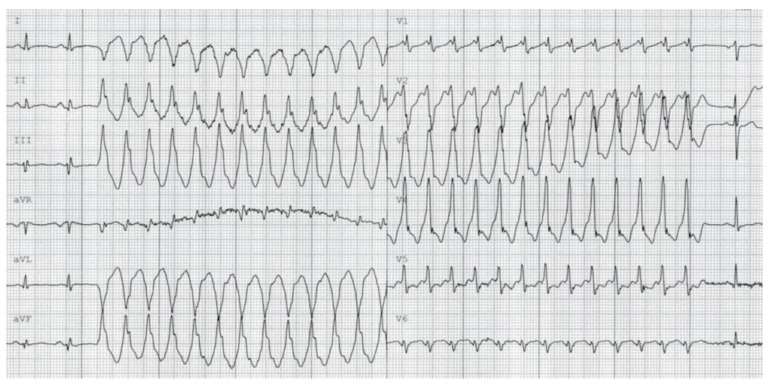
ECG showing a non-sustained VT during monitoring at the cardiac care unit (CCU).

Thirteen years after the initial presentation, the patient again experienced recurrent VTs (Figure 4), resulting in electrical storm, as well as concomitant amiodarone-induced hyperthyroidism. Consequently, the patient was treated with anti-thyroid medication (thiamazole and perchlorate) and underwent five sessions of plasmapheresis to lower fT4 levels. After the patient’s fT4 levels had returned to a normal range, a thyroidectomy was scheduled. Furthermore, an electrophysiological study (EPS) with endocardial ablation was performed, which unfortunately failed to successfully prevent VT re-induction.

**Figure 5:**
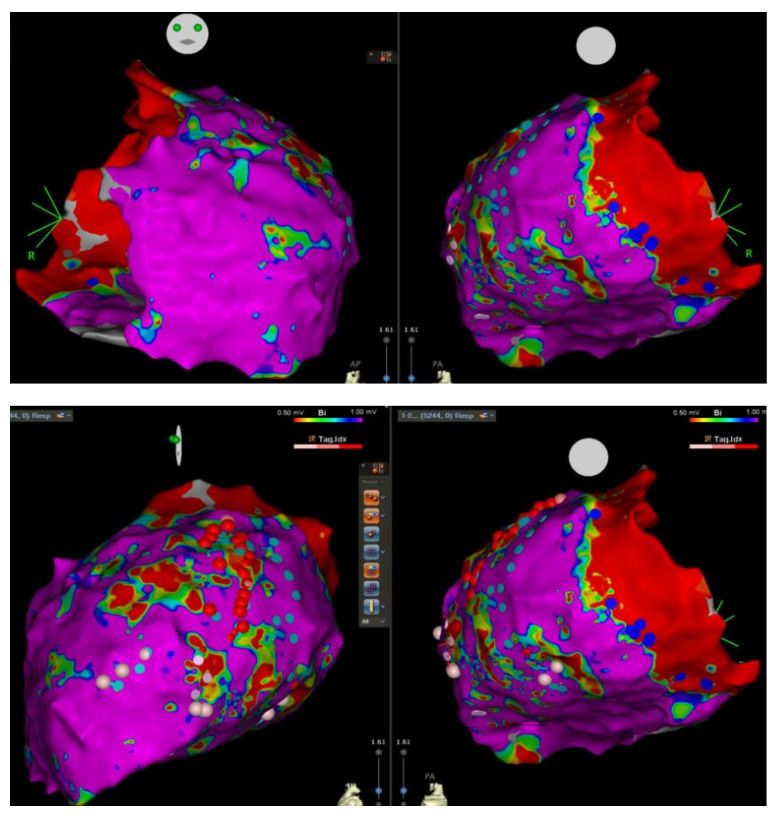
Epicardial high-density CARTO^®^ voltage map. The lower panels show multiple ablation points (red and pink circles).

Finally, three years after the failed endocardial ablation and sixteen years after the initial presentation, successful VT epicardial ablation was performed (Figure 5): in sinus rhythm, the epicardial mapping showed a large ventricular scar area with reduced voltage and very late potentials. At one site with very late potentials, ventricular stimulation induced a VT resembling clinical VT with very wide ventricular complexes. A total of 88 late potentials in the epicardium were ablated during the procedure (Figure 5, lower panels). The procedure was complicated by iatrogenic palsy of the left phrenic nerve which has not caused any symptoms for the patient to date.

## 7. Outcomes

No further arrhythmias were detected by the ICD (using remote monitoring) since successful epicardial VT ablation after a follow-up of three years.

## 8. Discussion

Physical exercise is widely acknowledged as one of the most effective tools for improving health. Interventions to reduce a sedentary lifestyle should, however, be accompanied with preventive measures and screening programs. Common causes of SCD in young athletes below the age of 35 years include structural cardiac abnormalities (hypertrophic cardiomyopathy, arrhythmogenic right ventricular cardiomyopathy, congenital coronary artery anomalies, Marfan syndrome, aortic stenosis), electrical cardiac abnormalities (Wolff Parkinson White syndrome, long QT syndrome, Brugada syndrome, catecholaminergic polymorphic ventricular tachycardia) or acquired cardiac abnormalities (myocarditis, commotio cordis, drug toxicity, hypo/hyperthermia) [4]. Beyond medical history and physical examination, diagnostic tools include imaging (echocardiography, CMR), different forms of exercise stress testing [5], genetics and electrophysiological studies. The single most important source for risk assessment is, however, the 12-lead resting ECG. Notably, placing the right precordial leads up to the second and third intercostal space increases sensitivity when diagnosing Brugada syndrome [6]. A high burden of premature ventricular complexes (PVCs) may prompt further investigations, focusing on the characteristics of the PVCs (monomorphic/polymorphic) and their response to exercise [7].

Commotio cordis (CC) is defined as the acute onset of VF in the setting of a blunt, nonpenetrating and relatively innocent blow to the anterior chest wall in the precordial region. VF occurs in the absence of cardiac structural injury or histologic damage [8,9]. While blunt thoracic trauma is common, the incidence of resulting CC is low. However, in recreational and competitive sports, CC is one of the most common causes of SCD [10]. In contrast, high-impact blows that result in traumatic damage to the heart and the overlying thorax are classified as cardiac contusion [11].

Hence, CC seems to be a primary electrical event resulting in the induction of VF. An experimental model on CC verified that VF is an underlaying arrhythmia induced by a chest blow [12]. Whether a chest blow results in VF depends on several variables, including location, velocity, shape of the object and, most importantly, the timing of the impact. VF is initiated when the blow occurs in an electrically vulnerable period of repolarization during the 20–40 milliseconds window on the upslope of the T-wave. The rapid rise in left ventricular pressure leads to myocardial stretch, activating ion channels including the K^+^_ATP_ channel (mechano-electric coupling) and augmentation of repolarization, and causing premature ventricular depolarization, triggering VF. In the swine model, pharmacological inhibition of K^+^_ATP_ channels prior to the chest wall impact leads to a significant decrease in VF-induction [13]. Additionally, not all chest wall impacts produce VF. A total of 70% of impacts that do not result in VF but result in premature ventricular contraction without lethal arrhythmia induction [4]. Therefore, VF induction seems not only to depend on the mechano-electric trigger, but the presence of an appropriate substrate to sustain VF is required as well. The upslope of the T-wave is a period of increased dispersion of myocardial repolarization. Interestingly, mechanical triggers that induce VF further increase repolarization dispersion. Consequently, chest impacts are not only the trigger for VF induction; they also might contribute to the creation of an optimal substrate to induce VF. In the swine model, repolarization abnormalities (manifested by QT-prolongation) contribute to the susceptibility to chest-trauma-induced VF [13]. Other arrhythmias and conduction abnormalities, such as premature ventricular contractions, temporary heart block or bundle-branch block, could be induced by chest impacts at other time periods of the cardiac cycle [14,15].

Before 1995, the survival rate of CC in the U.S. Commotio Cordis Registry was only about 10%. A good outcome was only achieved by prompt cardiopulmonary resuscitation by bystanders, starting within the first minute. Recent data show survival rates that exceed 50%. The improved survival rates can be attributed to various factors, including increased awareness and identification of CC, leading to a decreased time period between collapse and cardiopulmonary resuscitation and defibrillation. Furthermore, the availability of automated external defibrillators in the community and an increase in the number of individuals trained and willing to perform these life-saving interventions have also contributed to the improved outcomes [9,16].

The presented patient was rather old as the mean age of CC patients is 14 years [17]. This might be due to a higher chest compliance in children [12] as well as an increased likelihood of participation in implicated sports.

This case report is particularly interesting, as most CC victims are hit during a baseball, softball, or ice hockey match [18], but rarely while playing soccer. In fact, softer objects are associated with a decreased risk of VF induction. Compact and small objects (such as a golf ball) induce VF more frequently than larger objects, which distribute the energy of impact over a wider area [12,19]. This highlights the importance of the size and shape of the object as a risk factor for CC.

To induce VF in commotio cordis, blows must be delivered to the precordial chest wall at a direct perpendicular angle. The ideal impact velocities to cause CC are slightly lower than those that cause cardiac damage, with an optimal velocity of 65 km/h in the swine model, while a velocity of 80 km/h results in cardiac damage [20].

Monomorphic ventricular tachycardia due to electrical and structural ventricular remodeling long after a blunt chest trauma is a very rare manifestation and mostly occurs due to ventricular aneurysm and/or tissue fibrosis that develops gradually after the initial injury. If patients develop an abnormal ventricular substrate leading to VT beyond the acute injury phase, an aggressive management and treatment approach is required, as seen in our case [21,22].

## 9. Conclusions

This case shows another non-penetrating blunt thoracic trauma inducing sudden cardiac arrest due to ventricular fibrillation, as well as late-onset recurrent VT due to delayed electrical and structural ventricular remodeling, which highlights the importance of ICD implantation in such cases.

## Figures and Tables

**Table 1:** Timeline.

Time zero, aged 32 years	Commotio cordis while playing soccer with survived cardiac death (ventricular fibrillation)
First month after CC	Recovery and implantation of VVI-ICD
Two years later	Inappropriate ICD shocks due to Riata^®^ electrode malfunction; implantation of a new ICD lead without removal of the old one
Six years later	First appropriate ICD shock due to fast VT, repeated during playing soccer (performed against our advice)
Thirteen years later	Unsuccessful endocardial VT ablation attempt
Sixteen years later	Primarily successful VT epicardial ablation, complicated by iatrogenic palsy of the left phrenic nerve

## Data Availability

All data are available on a reasonable request to the corresponding author.
